# Two-stage group-sequential designs with delayed responses – what is the point of applying corresponding methods?

**DOI:** 10.1186/s12874-024-02363-7

**Published:** 2024-10-17

**Authors:** Stephen Schüürhuis, Gernot Wassmer, Meinhard Kieser, Friedrich Pahlke, Cornelia Ursula Kunz, Carolin Herrmann

**Affiliations:** 1 Institute of Biometry and Clinical Epidemiology, Charité - Universitätsmedizin Berlin, Corporate member of Freie Universität Berlin and Humboldt-Universität zu Berlin, Institute of Biometry and Clinical Epidemiology, Charitéplatz 1, Berlin, 10117 Germany; 2 RPACT GbR, Am Rodenkathen 11, Sereetz, 23611 Germany; 3 Institute of Medical Biometry, University Medical Center Ruprechts-Karls University Heidelberg, Im Neuenheimer Feld 130.3, Heidelberg, 69120 Germany; 4 Biostatistics and Data Sciences, Boehringer Ingelheim GmbH & Co. KG, Birkendorfer Straße 65, Biberach an der Riß, 88400 Germany; 5 Mathematical Institute, Heinrich Heine University Düsseldorf, Düsseldorf, Germany

**Keywords:** Group-sequential design, Delayed response, Pipeline data, Performance evaluation, Conditional performance score, rpact

## Abstract

**Background:**

In group-sequential designs, it is typically assumed that there is no time gap between patient enrollment and outcome measurement in clinical trials. However, in practice, there is usually a lag between the two time points. This can affect the statistical analysis of the data, especially in trials with interim analyses. One approach to address delayed responses has been introduced by Hampson and Jennison (J R Stat Soc Ser B Stat Methodol 75:3-54, 2013), who proposed the use of error-spending stopping boundaries for patient enrollment, followed by critical values to reject the null hypothesis if the stopping boundaries are crossed beforehand. Regarding the choice of a trial design, it is important to consider the efficiency of trial designs, e.g. in terms of the probability of trial success (power) and required resources (sample size and time).

**Methods:**

This article aims to shed more light on the performance comparison of group sequential clinical trial designs that account for delayed responses and designs that do not. Suitable performance measures are described and designs are evaluated using the R package rpact. By doing so, we provide insight into global performance measures, discuss the applicability of conditional performance characteristics, and finally whether performance gain justifies the use of complex trial designs that incorporate delayed responses.

**Results:**

We investigated how the delayed response group sequential test (DR-GSD) design proposed by Hampson and Jennison (J R Stat Soc Ser B Stat Methodol 75:3-54, 2013) can be extended to include nonbinding lower recruitment stopping boundaries, illustrating that their original design framework can accommodate both binding and nonbinding rules when additional constraints are imposed. Our findings indicate that the performance enhancements from methods incorporating delayed responses heavily rely on the sample size at interim and the volume of data in the pipeline, with overall performance gains being limited.

**Conclusion:**

This research extends existing literature on group-sequential designs by offering insights into differences in performance. We conclude that, given the overall marginal differences, discussions regarding appropriate trial designs can pivot towards practical considerations of operational feasibility.

**Supplementary Information:**

The online version contains supplementary material available at 10.1186/s12874-024-02363-7.

## Introduction

Pipeline data are known to be a problem in trials with time-to-event data but they can also cause issues in trials with continuous or categorical endpoints if the outcome is only available after some time lag. Many clinical trial designs for continuous or binary endpoints assume that the outcome measurement is immediately available once the patient has been recruited. In cases where interim results are used to make modifications or decide on the trial continuation, a potential outcome delay needs to be taken into consideration. Most group-sequential and also adaptive design methodology does not consider explicitly this potential delay in the availability of the outcome measurement, while especially group-sequential trial designs are frequently applied, e.g., [1]. Tsiatis and Davidian [2] state: “Typically, the interim analysis is based only on the data from subjects for whom the outcome has been ascertained”. Nevertheless, this approach not only neglects the potential for obtaining more robust evidence but also gives rise to ethical and regulatory concerns. The ethical concern arises from treating subjects without subsequently using their data, posing a potential conflict with regulatory guidelines, such as those stipulated by the European Medicines Agency (EMA) [3], which demands the use of all patient data. As a consequence, effort has been put into the development of methods that aim at formally incorporating the pipeline data into a testing strategy in order to comply with the requirement to use all data for testing. Different methods that address the issue of incorporating pipeline patients into testing strategies have been proposed in earlier work [4–7].

Note that we use the term delayed responses synonymously to time-lagged outcome where the delay is associated with the outcome itself. However, we define overrunning as the additional data collection, e.g., due to ongoing randomization during an interim analysis. There exists separate literature on the topic of overrunning [8–10], where, e.g., the deletion method [5] is known for long but also to be not that powerful. The method by Whitehead describes calculating the boundary for significance testing using the overrunning data while ignoring the data based on which the recruitment has been terminated beforehand [5]. Other methods involve p-value combination tests to combine data from before and after stopping recruitment [10, 11].

Schmidt et al. [6] consider the problem of overrunning in case of sequential trials with discrete data and multiple hypotheses. They address the challenge of incorporating pipeline patients into the testing strategy, particularly when some hypotheses have already been rejected without considering pipeline data, while others remain unrejected (at least) until that data becomes available. To address this, they propose a solution that requires a “second rejection” of the previously rejected hypotheses, now including the pipeline patients. Their approach is based on the concept of conditional error functions. Furthermore, the EMA includes a section on overrunning in their reflection paper on methodological issues in confirmatory clinical trials planned with an adaptive design [3]. They comment on primary endpoints not being immediately observed or a continuation of randomization during the interim analysis and state that the decision needs to be based on the final results of the trial. Mehta and Pocock [12] propose the “promising zone approach” for sample size recalculation in their article and compare the new design considering the pipeline patients against ignoring them. However, they do not formally incorporate those pipeline patients into the design but simply add them to the expected sample size. Due to the therefore too conservative interim boundaries and thus a low probability for stopping early, the impact of pipeline patients on the expected sample size is quite small. Jennison and Turnbull [7] extended the work by Mehta and Pocock [12]. In addition to introducing different ways of adapting the sample size, they apply these methods to an example with pipeline data. Jennison and Turnbull do not compare error-spending group-sequential designs and delayed response group-sequential designs. Their focus lies on the presentation of optimal solutions but less on whether design complexity that is introduced by pipeline data can justify a potential performance gain. Hampson and Jennison [4] proposed several delayed response group-sequential designs that incorporate pipeline data into a testing strategy. Their general idea is to split the interim decision on an irreversible recruitment stop and a subsequent hypothesis test in case stopping was indicated. They consider both error- spending tests and the fraction of information in pipeline and compare optimal delayed response group-sequential designs and standard group-sequential designs with immediate response with respect to the expected sample size. They do not compare the delayed response error spending designs to a standard group-sequential error spending design.

The topic of pipeline data in group-sequential trial designs is still a topic of ongoing discussion. Tsiatis and Davidian [2] suggested a general group-sequential framework for clinical trials with delayed response outcomes that accounts for baseline and time-dependent covariates. They show that the associated test statistics maintain the independent increments structure. Schüürhuis et al. [13] recently introduced a group-sequential design that integrates delayed responses, focusing on the possibility of reopening a trial after a potential intermediate pause. Their approach involves establishing recruitment stopping boundaries based on the probability of halting the trial for efficacy once the pipeline data becomes available, i.e. the interim conditional power. Mukherjee et al. [14] considered delayed response in two-stage single-arm trials (referring usually to phase 2 trials). Recently, they also conducted research on two-arm trials [15]. They did an in-depth analysis of the impact of delayed responses when standard group-sequential methods are applied. However, they did not consider group-sequential methods developed for delayed responses and their work can therefore be seen as an extensive diagnosis of pitfalls of standard group-sequential methods when delayed responses are prominent.

The goal of this paper is a more holistic evaluation of delayed response designs than it can be found in the literature so far. Especially, we are interested in whether a potential gain in performance can justify the use of more complex designs addressing pipeline data. Moreover, we want to evaluate the influence of the amount of pipeline data. Hence, it is crucial to define appropriate performance criteria. Standard effect measures are the average sample size, (conditional) power as well as early stopping probabilities. We apply the delayed response group-sequential methods as they have been newly implemented in the R package rpact [16].

The structure of the manuscript is as follows: After a brief recapitulation of group-sequential designs we describe the methods used for dealing with delayed responses, as well as give some background on performance evaluation. As our primary objective is to emphasize designs that are both practically relevant and commonly applied, our paper concentrates on nonbinding stopping boundaries, which are preferred and recommended over their binding counterparts [17, 18]. Afterwards, we present the results of an extensive comparative study evaluating the performance gain of statistical methods accounting for delayed responses over standard group-sequential methods as well as the influence of the amount of pipeline data. We close with a discussion where we also give further insights into the practical applicability of the performance measures used in the manuscript with the R package rpact.

## Group sequential designs

We consider two-stage group -sequential study designs including one interim analysis, with two equally sized arms of sample size *n* (in the intervention group *I* and control group *C*). More precisely, we assume the endpoint to be normally distributed

$$\begin{aligned} \begin{array}{l} X_{i,C} \sim N(\mu _C, \sigma ^2),\\ X_{i,I} \sim N(\mu _I, \sigma ^2), i = 1, ..., n,\\ \end{array} \end{aligned}$$

with common but unknown standard deviation $\sigma$. We test the one-sided hypotheses

$$\begin{aligned} \mathcal {H}_0: \mu _I - \mu _C \le 0 \text { versus } \mathcal {H}_1: \mu _I - \mu _C > 0. \end{aligned}$$

Due to the group -sequential design, the hypothesis is tested after $n_1<n$ patients per group have been included into the first stage and potentially also after a second stage. Continuation with the second stage takes place only if the trial was not stopped for efficacy or futility at the interim analysis. The interim test statistic is given by

$$\begin{aligned} Z_1 = \frac{\bar{X}_{{\textbf {1}}, I}- \bar{X}_{{\textbf {1}}, C}}{S_{pool, {\textbf {1}}}} \cdot \sqrt{\frac{n_1}{2}}, \end{aligned}$$

with means $\bar{X}_{{\textbf {1}}, I}$ and $\bar{X}_{{\textbf {1}}, C}$ as well as pooled standard deviation $S_{pool, {\textbf {1}}}$. The trial stops for efficacy if $Z_1 \ge u_1$ and for futility if $Z_1 \le l_1$, where $u_1$ and $l_1$ represent the upper and lower boundaries of the continuation region. In conventional group-sequential designs, these critical values can be calculated as quantiles from a multivariate normal distribution. Corresponding local significance levels can be determined based on $\alpha$-spending functions [19], such as those mimicking the boundaries proposed by O’Brien Fleming [20] or Pocock [21] (see Appendix A). Whenever the first stage test statistic falls between $l_1$ and $u_1$, the trial is continued with a second stage where a separate test statistic is calculated:

$$\begin{aligned} Z_2 = \frac{\bar{X}_{{\textbf {2}}, I}- \bar{X}_{{\textbf {2}}, C}}{S_{pool, {\textbf {2}}}} \cdot \sqrt{\frac{n_2}{2}}. \end{aligned}$$

Note that $Z_2$ consists solely of the second-stage data, i.e. $n_2=n-n_1$ additionally recruited patients per group. The final test decision is based on the combination of the first and second stage data, given here by the inverse normal combination test [22]:

$$\begin{aligned} Z_{1+2} = \frac{w_1 Z_1 + w_2 Z_2}{\sqrt{w_1^2 + w_2^2}}, \end{aligned}$$

where $w_1, w_2$ are weights which were defined at the planning stage of the trial. Throughout this paper, the weights are set as $w_1 = \sqrt{\frac{n_1}{n}}$ and $w_2 = \sqrt{\frac{n_2}{n}}$, ensuring that $Z_{1+2}$ simply corresponds to a t-test statistic based on all trial data. The null hypothesis is finally rejected if

$$\begin{aligned} Z_{1+2} \ge u_2, \end{aligned}$$

where $u_2$ denotes the critical value at the second study stage. Instead of fixing an interim sample size at $n_1$, group-sequential designs can also be characterized by the information rate $I_1 = \frac{n_1}{n}$, which denotes the fraction of observations available at interim per arm. In the evaluations presented in “Results” section, we adopt this parameterization as it aligns more naturally with the utilization of error-spending functions.

### Delayed response case

In the standard group-sequential design setting outlined in “Group sequential designs” section, an implicit assumption is that, upon conducting the interim analysis, data from all recruited patients is considered in the calculation of test statistics. However, practical scenarios may introduce a temporal gap of $\Delta _t$ between patient recruitment and the measurement of the primary outcome, attributed to factors such as multiple treatment applications or administrative delays. This is often referred to as “delayed responses”. Assuming successive entry of patients into the trial, the consequence is that a certain number of patients $n_{\Delta _t}$ (or, equivalently, a certain fraction of information $I_{\Delta _t}$) has been recruited, yet their data is unavailable for interim analysis. In the following, we consider the error-spending tests by Hampson and Jennison [4] who introduced a delayed response group-sequential test that considers pipeline data from the planning stage onward.

**Fig. 1:**
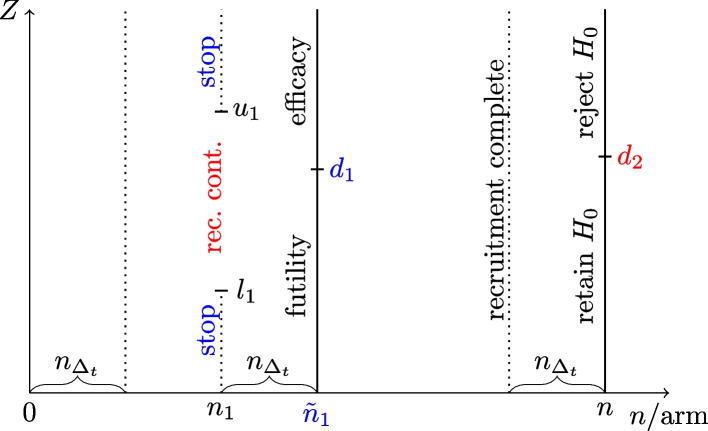
Structure of the delayed response group-sequential method by Hampson and Jennison [4]; x-axis: observation progress expressed in terms of sample size *n* per arm; y-axis: critical values on $Z-$scale; Different study paths, corresponding decision critical values $\{d_1, d_2\}$ and resulting sample sizes are highlighted in red (continue to final analysis) and blue (perform hypothesis test at interim)

Initially, delayed response group-sequential designs follow a similar process to standard group-sequential designs. That is, after observing $n_1 < n$ patients per group, the interim test statistic $Z_1$ is evaluated as before. In the delayed response setting, let now $n_{\Delta _t}$ represent the number of patients still in the pipeline upon observation of $Z_1$. We denote

$$\begin{aligned} \tilde{Z}_1 = \frac{\tilde{X}_{{\textbf {1}}, I}- \tilde{X}_{{\textbf {1}}, C}}{\tilde{S}_{pool, {\textbf {1}}}} \cdot \sqrt{\frac{\tilde{n}_1}{2}} \end{aligned}$$

as the test statistic after additionally observing the outstanding responses, signified by the use of a tilde, where $\tilde{n}_1 = n_1 + n_{\Delta _t}$. More specifically, $\tilde{X}_{{\textbf {1}}, I}$, $\tilde{X}_{{\textbf {1}}, C}$, and $\tilde{S}_{pool, {\textbf {1}}}$ represent the groupwise means and pooled standard deviation computed using the additional pipeline data. Similar to a standard group-sequential design, the vector of test statistics $\textbf{Z} = (Z_1, \tilde{Z}_1, Z_{1+2})^t$ is known to follow the canonical multivariate normal distribution, allowing the application of standard group-sequential design theory for the calculation of critical values and performance characteristics. As outlined in the previous descriptions, the method introduced by Hampson and Jennison splits the interim analysis into a decision on recruitment continuation, followed by a potential stop for efficacy. We represent the continuation region as the open interval $(l_1, u_1)$ and denote $\{d_1, d_2\}$ as the interim and final stage critical value for efficacy assessment. Note that $d_2$ becomes relevant only if recruitment is continued at the interim stage, i.e. if $Z_1 \in (l_1, u_1)$. Figure 1 presents an illustration of the study design. In this design, we observe efficacy at interim only if $Z_1 \notin (l_1, u_1)$ triggering a recruitment stop, followed by $\tilde{Z}_1 \ge d_1$. In other words, the mechanism leading to an interim efficacy stop differs between a delayed response design and a standard group-sequential design. As a result, the application of standard group-sequential boundaries to determine the boundary set $\{l_1, u_1, d_1, d_2\}$ is not straightforward. Hampson and Jennison made multiple proposals regarding the determination of those. Due to their similarity to standard group-sequential methods, we will present two error-spending versions in the following.

### Delayed response group-sequential design according to Hampson and Jennison (2013) with nonbinding lower boundary (DR-GSD)

Among other methods, Hampson and Jennison (2013) proposed employing error-spending theory to define the boundary set $\{l_1, u_1, d_1, d_2\}$ [4]. In their original publication, the approach is formulated such that the resulting lower boundary is binding. Since our focus is on designs with nonbinding boundaries, we demonstrate that their framework can also accommodate nonbinding lower boundaries. As done in their work, we start by defining $\{u_1, d_2\}$ as the solutions to

$$\begin{aligned} & P_{\delta =0}(Z_1 \ge u_1) = \alpha _1 \text { and} \\ & P_{\delta =0}(Z_1 < u_1, Z_{1+2} \ge d_2) = \alpha - \alpha _1, \end{aligned}$$

where $\alpha _1 < \alpha$ represents the fraction of type I error that is planned to be spent at interim. In order to obtain a lower boundary, it is additionally required that

$$\begin{aligned} & P_{\delta = \tilde{\delta }}(Z_1 \le l_1) = \beta _1 \text { and}\\ & P_{\delta = \tilde{\delta }}(Z_1 \in (l_1, u_1), Z_{1+2} < d_2) = \beta - \beta _1, \end{aligned}$$

where, analogically, $\beta _1 < \beta$ is the fraction of type II error to be spent at interim and $\tilde{\delta }$ denotes an assumption on the unknown true effect. Since the type I error rate is spent assuming to continue the trial after observing $Z_1 < u_1$, the resulting boundary $l_1$ will be nonbinding. If $\alpha _1$ and $\beta _1$ are obtained from standard error-spending designs, $\{u_1, l_1, d_2\}$ are simple error-spending boundaries. In the design proposed by Hampson and Jennison [4], the interim test for significance occurs only when the recruitment has been stopped beforehand, while the amount of pipeline data does not necessarily need to be known upon recruitment termination. Hence, the type I error rate can be written as

$$\begin{aligned} P_{\delta =0}(Z_1 \notin (l_1, u_1), \tilde{Z}_1 \ge d_1) + P_{\delta =0}(Z_1 \in (l_1, u_1), Z_{1+2} \ge d_2), \end{aligned}$$

To fully exhaust $\alpha$, they proposed determining $d_1$ by solving the equation

1 $$\begin{aligned} P_{\delta =0}(Z_1 \ge u_1, \tilde{Z}_1 < d_1) = P_{\delta =0}(Z_1 \le l_1, \tilde{Z}_1 \ge d_1). \end{aligned}$$

This aims to balance the probability of achieving promising results with $Z_1$ while subsequently accepting $\mathcal {H}_0$, with the probability of halting recruitment due to non-promising results with $Z_1$, followed by rejecting $\mathcal {H}_0$ afterwards under $\mathcal {H}_0$. With

$$\begin{aligned} P_{\delta =0}(Z_1 \notin (l_1, u_1), \tilde{Z}_1 \ge d_1) & = P_{\delta =0}(Z_1 \ge u_1, \tilde{Z}_1 \ge d_1) + P_{\delta =0}(Z_1 \le l_1, \tilde{Z}_1 \ge d_1) \\ & {\mathop {=}\limits ^{(1)}} P_{\delta =0}(Z_1 \ge u_1, \tilde{Z}_1 \ge d_1) + P_{\delta =0}(Z_1 \ge u_1, \tilde{Z}_1 < d_1) \\ & = P_{\delta =0}(Z_1 \ge u_1) = \alpha _1, \end{aligned}$$

we can immediately see that constraint (1) leads to type I error rate control if $l_1$ was a binding boundary. Since

$$\begin{aligned} P_{\delta =0}(Z_1 \notin (-\infty , u_1), \tilde{Z}_1 \ge d_1) \le P_{\delta =0}(Z_1 \notin (l_1, u_1), \tilde{Z}_1 \ge d_1) \le \alpha _1, \end{aligned}$$

type I error rate control is also guaranteed when declaring the $l_1$ to be nonbinding, demonstrating that the original Hampson and Jennison design also covers nonbinding lower recruitment stopping boundaries.

Such a delayed response design allows for $\mathcal {H}_0$ rejection, even if recruitment has been stopped due to undesirable values of $Z_1$. To account for this consideration, a revised delayed response design, maintaining the same underlying structure, has been suggested [23]. In this adaptation, boundary determination ensures that the design precludes a rejection decision after halting recruitment upon crossing $l_1$.

### Repeated rejection group-sequential design (RR-GSD)

In the preceding section, we illustrated that the design proposed by Hampson and Jennison [4] can also accommodate nonbinding lower recruitment stopping boundaries. However, this design permits the rejection of the null hypothesis at interim stages following recruitment halt due to non-promising results. A modified version for determining $\{u_1, l_1, d_1, d_2\}$ was proposed by Jennison and Hampson [23] posing the following constraints:

2 $$\begin{aligned} & P_{\delta =0}(Z_1 \ge u_1, \tilde{Z}_1 \ge d_1) = \alpha _1, \nonumber \\ & P_{\delta =0}(Z_1< u_1, Z_{1+2} \ge d_2) = \alpha - \alpha _1, \nonumber \\ & P_{\delta = \tilde{\delta }}(Z_1 \le l_1 \cup (Z_1 \ge u_1 \cap \tilde{Z}_1 < d_1)) = \beta _1, \end{aligned}$$

see also Schüürhuis et al. [13]. With the restriction that $l_1 < u_1$, we can rewrite Eq. (2) as

$$\begin{aligned} & P_{\delta = \tilde{\delta }}(Z_1 \le l_1 \cup (Z_1 \ge u_1 \cap \tilde{Z}_1< d_1)) \\ = & P_{\delta = \tilde{\delta }}(Z_1 \le l_1) + P_{\delta =\tilde{\delta }}(Z_1 \ge u_1 \cap \tilde{Z}_1 < d_1), \end{aligned}$$

showing that these equations can be solved numerically using the multivariate normal distribution presented in “Delayed response case” section [13]. Given $\{u_1, l_1, d_1, d_2\}$, we have three equations as given by (2), but four unknown boundaries. Jennison and Hampson [23] therefore suggested fixing $d_1 = \Phi ^{-1}(1-\alpha )$ and solving the equations above for $\{l_1, u_1, d_2\}$ iteratively. With this method, it is no longer permitted to reject at interim after observing $Z_1 \le l_1$ since the type I error rate is spent with regards to crossing the upper boundaries $\{u_1, d_1\}$ only. Put differently, the null hypothesis can only be rejected if positive outcomes are observed during both, the recruitment interruption and in the subsequent statistical test. Moreover, the lower recruitment stopping boundary $l_1$ can be considered nonbinding, as $d_2$ is calculated assuming to proceed with the trial after observing $Z_1 < u_1$. Note that, similar to a standard group-sequential design, crossing $l_1$ can be directly interpreted as an observation of futility, in contrast to the DR-GSD.

## Performance evaluation

When comparing different design options and judging how well a specific design performs, precise performance criteria need to be specified. Up to now, standard evaluation measures, such as average sample size or overall power, are nearly always applied. However, there is no mutual agreement upon which set of criteria should be used when comparing such different designs. In general, there exist two different points of views for performance evaluation. Both the global and the conditional perspective are valid and focus on a different aspect.

### Global performance criteria

The global evaluation perspective refers to the situation of evaluating the study design no matter whether the trial stops early at the interim analysis or it continues with the second stage. Standard measures in the non-delayed response setting are the expected overall sample size

$$\begin{aligned} \mathbb {E}[N] = n_1 + n_2(Z_1), \end{aligned}$$

where

$$\begin{aligned} n_2(Z_1) = \left\{ \begin{array}{ll} \,\, 0 & \quad \text {if}\quad Z_1 \in (l_1, u_1), \\ \,\, n_2 & \quad \text {if}\quad Z_1 \notin (l_1, u_1) \end{array}\right. \end{aligned}$$

is a random variable depending on $Z_1$ and the global power

$$\begin{aligned} Pow = \mathbb {P}_{\delta =\tilde{\delta }} (Z_1 \ge u_1) + \mathbb {P}_{\delta =\tilde{\delta }}\left( Z_{1+2} \ge u_2 \cap Z_1 \in (l_1, u_1)\right) , \end{aligned}$$

referring to the probability of correctly rejecting the null hypothesis either at the interim or the final analysis. Furthermore, the probability for an early efficacy and futility stop

$$\begin{aligned} \mathbb {P}_{\delta =\tilde{\delta }}(Z_1 \ge u_1) \quad \text {and} \quad \mathbb {P}_{\delta =\tilde{\delta }}(Z_1 \le l_1) \end{aligned}$$

are often reported.

When designs that account for delayed responses are applied, the expected sample size incorporates the pipeline data as well leading to

$$\begin{aligned} \mathbb {E}_{DR}[N] = n_1 + n_{\Delta _t} + n_2(Z_1), \end{aligned}$$

and the power of the DR-GSD is given by

$$\begin{aligned} Pow_{DR} = P_{\delta =\tilde{\delta }}(Z_1 \notin (l_1, u_1), \tilde{Z}_1 \ge d_1) + P_{\delta =\tilde{\delta }}(Z_1 \in (l_1, u_1), Z_{1+2} \ge d_2). \end{aligned}$$

Correspondingly, the early efficacy and futility stopping probability for delayed response group sequential designs is given by

$$\begin{aligned} P_{\delta =\tilde{\delta }}(Z_1 \notin (l_1, u_1), \tilde{Z}_1 \ge d_1) \quad \text {and} \quad P_{\delta =\tilde{\delta }}(Z_1 \notin (l_1, u_1), \tilde{Z}_1 < d_1). \end{aligned}$$

Note that that there exist several approaches for combining global performance measures into a single value in the literature, e.g. outweighing the global power against the average sample size [7]. A summary of expressions for the global performance criteria for the different designs introduced in “Delayed response case” section can be found in Table 1.

**Table 1:** Global performance characteristics for the GSD, DRGSD and DRD under consideration of pipeline data; Note that the boundary sets between those methods differ, see “Delayed response case” section

Design	*P*(*futility*)	$P(reject \, at \, interim)$	Power
GSD	$\mathbb {P}_{\delta =\tilde{\delta }}(Z_1 \le l_1)$	$\mathbb {P}_{\delta =\tilde{\delta }}(Z_1 \ge u_1)$	$\mathbb {P}_{\delta =\tilde{\delta }}(Z_1 \ge u_1) +$
			$$\mathbb {P}_{\delta =\tilde{\delta }}\left( Z_{1+2} \ge u_2 \cap Z_1 \in (l_1, u_1)\right)$$
DR-GSD	$P_{\delta =\tilde{\delta }}(Z_1 \notin (l_1, u_1), \tilde{Z}_1 < d_1)$	$P_{\delta =\tilde{\delta }}(Z_1 \notin (l_1, u_1), \tilde{Z}_1 \ge d_1)$	$P_{\delta =\tilde{\delta }}(Z_1 \notin (l_1, u_1), \tilde{Z}_1 \ge d_1) +$
			$$P_{\delta =\tilde{\delta }}(Z_1 \in (l_1, u_1), Z_{1+2} \ge d_2)$$
RR-GSD	$P_{\delta =\tilde{\delta }}(Z_1 \le l_1 \cup (Z_1 \ge u_1 \cap \tilde{Z}_1 < d_1))$	$P_{\delta =\tilde{\delta }}(Z_1 \ge u_1, \tilde{Z}_1 \ge d_1)$	$P_{\delta =\tilde{\delta }}(Z_1 \ge u_1, \tilde{Z}_1 \ge d_1) +$
			$$P_{\delta =\tilde{\delta }}(Z_1 \in (l_1, u_1), Z_{1+2} \ge d_2)$$

Abbreviations: *GSD* Group-sequential design, *DR-GSD* Delayed response group-sequential design, *RR-GSD* Repeated rejection group-sequential design

### A note on conditional performance criteria

The other point of view for comparing the designs is the conditional perspective, and usually it is recommended to evaluate a design from both the global and conditional perspective. Evaluating group-sequential or adaptive designs conditionally involves considering only cases where the interim test statistic suggests no early stopping for efficacy or futility. Here, this means that performance criteria such as power and expected sample size are conditional on having observed some $Z_1 \in (l_1, u_1)$.

An example of a comprehensive conditional performance metric is the conditional performance score proposed by Herrmann et al. [24]. This score incorporates both the *conditional expected sample size* (*CN*) and the *conditional power* (*CP*). For both metrics, measures of location and variation are considered, represented by their *expected values* and *variances*. For some details on the score, refer to Appendix B.

Since conditional performance measures involve conditioning on study continuation at interim, a fair comparison between designs is only possible when the continuation criteria are consistent. Given that we exclusively examine group-sequential trial designs with a fixed second-stage sample size of $n_2$, the conditional expected sample size is consistent across all designs whenever $Z_1 \in (l_1, u_1)$. Specifically, we have that $\mathbb {E}_{\delta=\tilde{\delta}} [N|Z_1 \in (l_1, u_1)]=n_1 + n_2$, and correspondingly, $Var_{\delta=\tilde{\delta}} [N|Z_1 \in (l_1, u_1)] = 0$. Therefore, when considering conditional sample size metrics, the comparison between the designs yields equal results. In contrast, the conditional power, defined as $CP~:=~P_{\delta=\tilde{\delta}} (Z_{1+2} \ge d_2|Z_1 \in (l_1,u_1))$, additionally depends on the critical value $d_2$. For DR-GSD and GSD, where $\{l_1, u_1, d_2\}$ are determined to be the same, the conditional power is identical for both designs. Comparisons with the RR-GSD are not reasonably interpretable due to differing continuation regions.

The conditional performance score introduced by Herrmann et al. [24] incorporates not only conditional performance metrics but also their deviation from target values. Appendix B and Appendix C summarize these target values, demonstrating that, as long as pipeline data is available, a standard group-sequential design not accounting for those does not differ in terms of target values from the delayed response model considered in this work. Consequently, aggregating conditional performance measures still yields equal performance, provided the continuation region and final stage critical value are the same across the designs under comparison. Overall, while the conditional performance score itself is a suitable tool for the evaluation of delayed response designs with the same continuation region, it does not provide sensible insights into performance comparison between a standard group-sequential design and a delayed response design. This is due to the fact that upon conditioning on continuation, the designs are equivalent. In conclusion, for the remainder of our work, we will focus solely on the performance difference from a global perspective.

## Results

As recommended in the preceding section, the performance assessment of diverse study designs can be conducted from both a global and a conditional perspective. In the following, we will employ only the global perspective to evaluate the designs outlined in “Group sequential designs” section, as conditional measures are not meaningful in this specific scenario. Our primary focus is on determining whether the formal integration of pipeline data into a testing strategy results in improved global performance. We will examine various scenarios detailed in Table 2. The spending functions are selected to mimic O’Brien-Fleming boundaries and Pocock boundaries, see Appendix A for details. The former boundaries exhibit monotonically decreasing upper limits, while the latter produce nearly constant critical values for efficacy assessment. Given that the error-spending proposal by Hampson and Jennison [4] incorporates $\beta$-spending for futility assessment, we will adopt a similar approach. Specifically, we will utilize $\beta$-spending functions aligned with the Pocock and O’Brien-Fleming $\alpha$-spending functions.

**Table 2:** Summary of the considered parameter constellations for performance comparison

Parameter	Values
Significance level $\alpha$	0.025
Type-II error $\beta$	0.2
Effect $\delta$	$[-0.4,0.8]$
Standard deviation $\sigma$	1
Maximum sample size per arm $n_{max}$	200
Interim information fraction $I_1$	$\{0.3,0.4,0.5\}$
Pipeline information fraction $I_{\Delta _t}$	$\{0.1,0.2,0.3\}$
Spending functions	O’Brien-Fleming-type spending, Pocock-type spending

Furthermore, our performance comparison will center on examining the impact of two key parameters: (1) the timing of the interim analysis, represented by the information fraction $I_1$, and (2) the fraction of information still in the pipeline, denoted as $I_{\Delta _t}$. This section begins with an evaluation of the aforementioned scenarios from a global perspective, that is, of power and expected sample size. As the designs differ particularly in terms of the mechanism that potentially leads to an interim efficacy or futility stop, we also examine the interim probability of stopping for efficacy or futility. For all evaluations, we make extensive use of the R package rpact (version 3.4.0) by Wassmer and Pahlke [16].

### Global performance

In this section, we provide the power and expected sample size evaluation for the scenarios listed in Table 2 for the Pocock-like and O’Brien-Fleming-like boundary design separately. Note that in standard group-sequential designs, although pipeline data are not used for decision-making, pipeline data are still included in the expected sample size calculation, as detailed in “Performance evaluation” section. In addition to power, we also provide results concerning the probability of early trial termination due to efficacy or futility. In that, the plots will all have the same structure, consisting of 3x3 grids. The columns contain the interim information fraction $I_1$ in increasing order, while the rows contain the amount of information fraction in pipeline $I_{\Delta _t}$, again ordered increasingly. This is done in order to illustrate the simultaneous impact of those parameters on performance differences. The *x*-axes represent the effect $\delta$, and the *y*-axes the probabilities and expected sample size values. Different designs correspond to different line colors as follows:

- Green: DR-GSD
- Yellow: GSD
- Blue: RR-GSD

For the plots containing probabilities, linetype is used as an additional tool to distinguish interim from overall power and futility from efficacy. For the expected sample sizes, we used different linetypes to highlight overlapping lines. Note that values of $\delta < 0$ are included to examine the probability to stop for futility.

#### Pocock-like boundaries

**Fig. 2:**
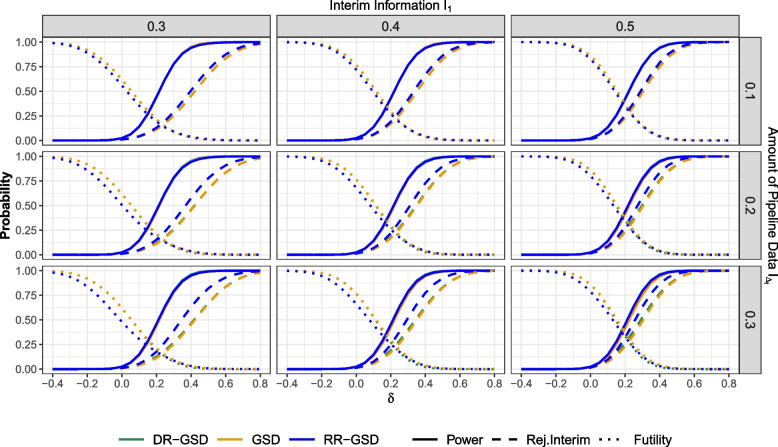
Probabilities for early stopping and power of the different considered designs at effect sizes $\delta$, depending on $I_1 \in \{0.3,0.4,0.5\}$ and $I_{\Delta _t} \in \{0.1,0.2,0.3\}$. The maximum sample size is set to $n_{max} = 200$ for both arms. Abbreviations: GSD: Group-sequential design; DR-GSD: Delayed response group-sequential design; RR-GSD: Repeated rejection group-sequential design

Figure 2 illustrates the power in the Pocock-like case for different information fraction settings. At first, the methods barely differ in power when the interim analysis occurs early and there is limited data in the pipeline (see e.g. upper left plot). This pattern is consistent for both total and interim power. Upon closer inspection, one can see that the DR-GSD has a slightly larger power than the other methods. That is, the delayed response method is slightly superior over the non-delayed response method in case futility is set up equivalently. As the amount of data in the pipeline increases, the RR-GSD demonstrates higher interim power compared to other methods due to its more liberal stopping criteria (see lower left plot). With $I_1 = 0.4$ and $I_{\Delta _t} = 0.2$, we find that the probability of rejecting $\mathcal {H}_0$ at interim is 0.496 for the GSD, 0.507 for DR-GSD, and 0.606 for RR-GSD when $\delta = 0.3$. This reflects an approximately $10\%$ increase in the probability to reject $\mathcal {H}_0$ at interim with the RR-GSD. Examining overall power, we observe 0.878 for the GSD, 0.889 for DR-GSD, and 0.882 for RR-GSD, suggesting minor differences in overall power within this setting. Concerning futility, the probability of stopping early is 0.069 for the GSD, 0.057 for the DR-GSD, and 0.069 for the RR-GSD, indicating a slightly smaller probability for DR-GSD. As $I_1$ increases, the differences between the methods become more pronounced, revealing that the overall power gain of delayed response methods can be attributed to a slightly larger interim power (see lower right plot). That is, the power of the delayed response design is particularly increased in regards to interim stopping, while global power is comparable across the whole range of effect sizes. Regarding the probability to stop for futility, we obtain similar curves for the DR-GSD and GSD especially under $\mathcal {H}_0$. On the other hand, we observe that the the RR-GSD has a decreased probability to stop for futility as compared to the other methods for effect sizes $\delta \lessapprox 0.2$. As the effect increases, the probability to stop for futility becomes the smallest for the DR-GSD.

**Fig. 3:**
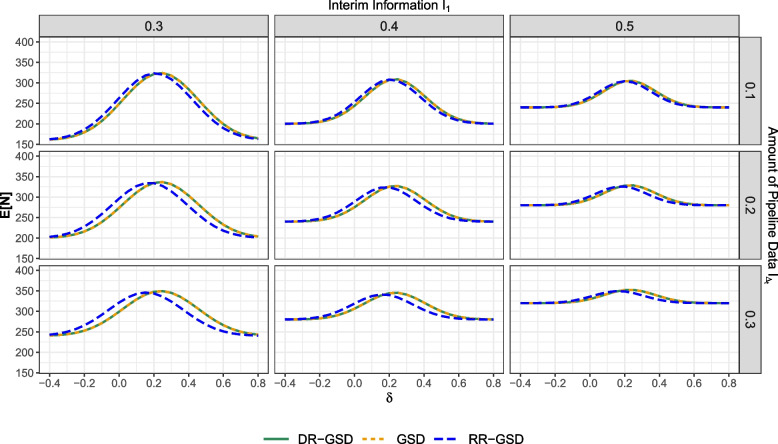
Total expected sample size $E_\delta [N]$ of the different considered designs at effect sizes $\delta$, depending on $I_1 \in \{0.3,0.4,0.5\}$ and $I_{\Delta _t} \in \{0.1,0.2,0.3\}$. The maximum sample size is set to $n_{max} = 200$ for both arms. Abbreviations: GSD: Group-sequential design; DR-GSD: Delayed response group-sequential design; RR-GSD: Repeated rejection group-sequential design

Figure 3 illustrates expected sample size values in the Pocock-like case. Notably, all designs result in sample size savings compared to a correspondingly planned single-stage design with an overall sample size of 400. Firstly, we observe that, for any setting, the DR-GSD and GSD methods do not differ in terms of expected sample size, indicated by the overlapping yellow and green lines. This similarity arises because the expected sample size is influenced by the probability of proceeding to the second stage $P_\delta (Z_1 \in (l_1, u_1))$, and $(l_1, u_1)$ is the same for both designs (see Table D1 in the supplementary material). Substantial differences are only evident when comparing RR-GSD with the other two designs (see e.g. lower left plot). For small or negative effect sizes, the RR-GSD yields larger expected sample sizes. However, for positive effect sizes of $\delta \gtrapprox 0.25$, the expected sample size values drops below those of the other designs. This effect is especially pronounced for small $I_1$ and large $I_{\Delta _t}$. For instance, consider a trial planned with $I_1 = 0.3$, while there is $I_{\Delta _t} = 0.3$ of information in pipeline, referring to to the lower left plot. In this scenario, the expected sample sizes for for $\delta = -0.1$ are as follows: GSD $= 271.1$, DR-GSD $= 271.1$, and RR-GSD $= 291.5$. However, for $\delta = 0.3$, we observe different values: GSD $= 345.2$, DR-GSD $= 345.2$, and RR-GDS $= 324.0$. Note that as $I_1$ increases (late interim) and $I_{\Delta _t}$ is smaller (low amount of pipeline data), the difference between the expected sample sizes diminishes. The observed pattern can be attributed to the following: In RR-GSD, the parameter $\beta _1$ influencing the determination of futility boundaries $l_1$ and $d_1$ is divided across two possibilities: - $Z_1 \le l_1$ or $Z_1 \ge u_1 \cap \tilde{Z}_1 < d_1$. Consequently, $l_1$ becomes conservative, making it less likely to stop for $Z_1 \le l_1$, resulting in a wide continuation region (see Table D1 in the supplementary material). Therefore, the trial is more likely to continue for small or negative effects, leading to a larger expected sample size. Conversely, this method allows for stopping for efficacy only when observing $Z_1 \ge u_1 \cap \tilde{Z}_1 \ge d_1$, whereas for DR-GSD, efficacy stop is concluded if $Z_1 \notin (l_1, u_1) \cap \tilde{Z}_1 \ge d_1$. Thus, the efficacy stopping criteria are more liberal, resulting in lower expected sample sizes for RR-GSD. This aligns with the increased probability of stopping at the interim, as shown in Fig. 2. The minimum sample size $\tilde{n}_1 = (I_1 + I_{\Delta _t})n_{max}$ is an increasing function in $I_1$ and $I_{\Delta _t}$. Hence, as $I_1$ and $I_{\Delta _t}$ increase, the minimum sample size $\tilde{n}_1$ also increases, such that we observe larger minimum values and a reduced value range in the bottom right corner of the plot grid.

#### O’Brien-Fleming-like boundaries

**Fig. 4:**
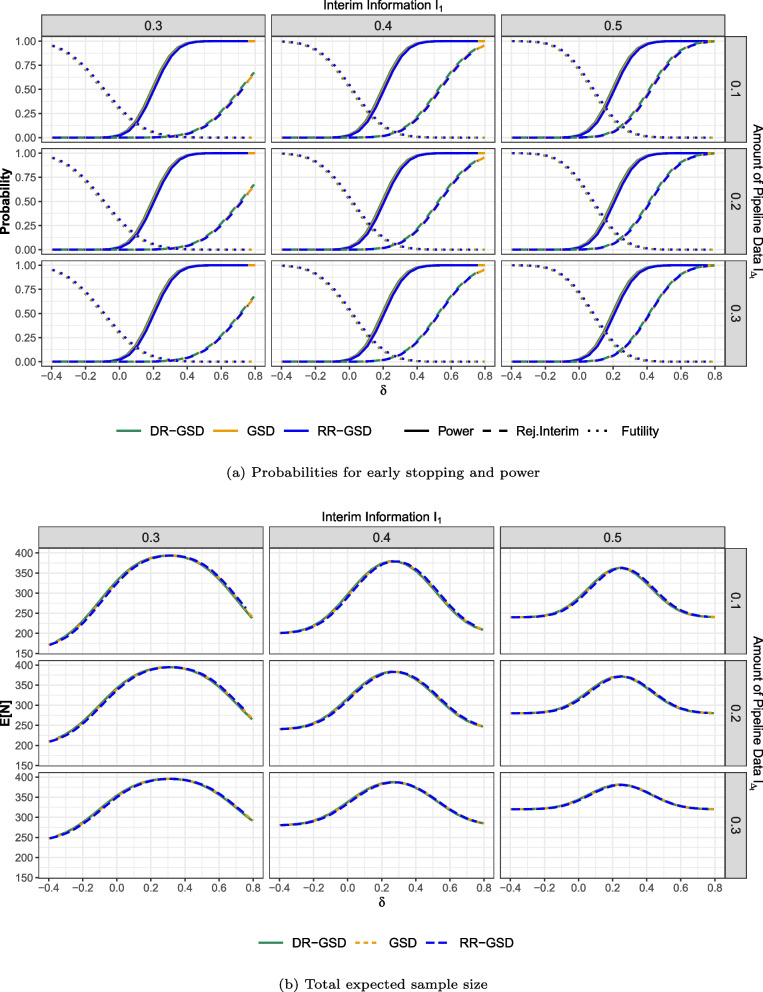
Probabilities for early stopping, power and total expected sample size $E_{\delta }[N]$ of the different considered designs at effect sizes $\delta$, depending on $I_1 \in \{0.3,0.4,0.5\}$ and $I_{\Delta _t} \in \{0.1,0.2,0.3\}$. Note that the curves of the different methods are all displayed, but mainly overlapping. Therefore, the lines are slightly shifted to make all lines visible. Abbreviations: GSD: Group-sequential design; DR-GSD: Delayed response group-sequential design; RR-GSD: Repeated rejection group-sequential design

In this section, we present analogous performance results for the O’Brien-Fleming-like case in Fig. 4, beginning with relevant probabilities (see (a)) and subsequently addressing expected sample size (see (b)).

In terms of power, the qualitative differences from the previous case are marginal. The observed differences are clearly less pronounced than in the Pocock-case. This is attributable to the generally more conservative nature of interim boundaries for efficacy with O’Brien-Fleming designs (see Table D1 in the supplementary material). In contrast to the previous case, where RR-GSD exhibited notably higher expected sample sizes for small and negative effects, this effect is no longer present. Again, this is due to the fact that the use of O’Brien-Fleming-like spending functions results in comparable boundary sets for the RR-GSD, whereas with the Pocock-like spending functions, the interim boundaries are notably different (see Table D1 in the supplementary material).

#### Power and expected sample size simulation

**Fig. 5:**
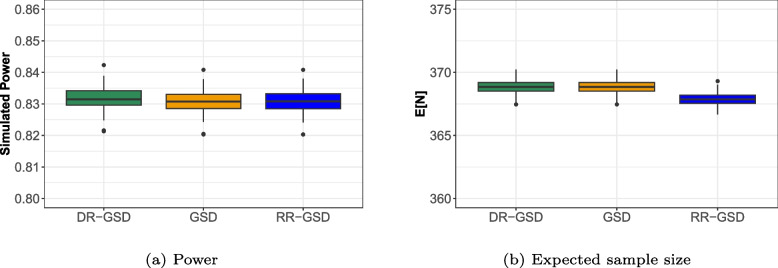
Boxplot of simulated power and expected sample size values for an O’Brien-Fleming design with $I_1 = 0.5$, $I_{\Delta _t} = 0.2$ at $\delta = 0.3$ and $\sigma =1$; Maximum sample size is $n_{\text {max}} = 200$ per Arm. Note that the differences may appear slightly more pronounced due to the narrow y-axis scaling but are actually negligible as also shown in Fig. 4. Abbreviations: GSD: Group-sequential design; DR-GSD: Delayed response group-sequential design; RR-GSD: Repeated rejection group-sequential design

In “Performance evaluation” section, we argued that a holistic performance evaluation should not only include location but also variation measures, given that performance characteristics inherently involve randomness that stems from random data. As closed-form formulas for the variance of power and expected sample size are not readily available, we employ simulations to assess the variability of the considered performance metrics. For computational efficacy, we simulated the test statistics directly, using the knowledge about their underlying multivariate normality. This simulation was implemented using the |mvtnorm| package (version 1.1-3) by Genz et al. [25]. We generated $nsim = 10,000$ test statistics for all stages and counted the number of rejections at interim and final stages, given that no interim stop occurred earlier. By repeating this procedure $k=100$ times, we obtained an empirical distribution of power and expected sample size values for the different designs shown in Fig. 5. Note that as the number of repetitions *k* increases, the distribution of values tends to center around the true value, resulting in smaller boxes in the boxplots. Therefore, to maintain clarity in discerning between the methods, we opted to set $k = 100$.

Figure 5 displays boxplots representing the empirical distributions of power and expected sample size values. The colors are again representative of the methods. The expected sample size is marginally smaller for the RR-GSD, aligning with the previous findings, while no notable power differences are apparent. Examining the variance of power between simulation runs, we observe small differences, as indicated by the y-axis ranging from 0.8 to 0.86. Similarly, the variance of the expected sample size is small, leading to the conclusion that none of the methods exhibits superiority in terms of the extent to which performance characteristics vary based on random data.

### Practical example

We illustrate various design approaches using the schizophrenia trial presented in Mehta and Pocock [12]. The trial is a two-arm randomized controlled phase III clinical trial comparing a verum drug against an active comparator drug in patients with negative symptoms of schizophrenia. The primary endpoint of this trial is measured using the Negative Symptoms Assessment Scale (NSA), as detailed by Alphs et al. [26]. This endpoint is considered a quantitative variable, with an assumed effect size of $\delta = 1.6$ and a standard deviation of $\sigma = 7.5$ in both groups.

To achieve a power of at least $80\%$ at a one-sided significance level of $\alpha = 0.025$, a fixed design would require a sample size per group of approximately

$$\begin{aligned} n = 2(z_{1-\alpha } + z_{1-\beta })^2\left( \frac{\sigma }{\delta }\right) ^2 \approx 345, \end{aligned}$$

resulting in a total sample size of $N = 690$. We will use this sample size for all of the four considered designs to compare power and expected sample size.

In Mehta and Pocock’s paper [12], they assume an average recruitment rate of 8 patients per week, leading to an overall recruitment period of approximately 86 weeks ($690/8 \approx 86$ weeks). Each patient requires $\Delta _t = 26$ weeks until the outcome can be ascertained, creating a delayed response situation. The authors propose an interim analysis after observing data from $n_1=200$ patients in total, which occurs after $(200/8+26)=51$ weeks. At this point, with $n_1 = 200$ observed patients, there are additional $n_{\Delta _t}=26\cdot 8 = 208$ patients in the pipeline, resulting in a total of $\tilde{n}_1 = 408$ patients. In terms of information rates, we hence have that $I_1 = 200/690 \approx 0.29$, and $\tilde{I}_1 = 408/690 = 0.59$. Table 3 below summarizes the planning assumptions.

**Table 3:** Summary of the considered parameter constellation for practical example

Parameter	Values
Effect $\delta$	1.6
Standard deviation $\sigma$	7.5
Maximum sample size per arm $n_{max}$	345
Interim information fraction $I_1$	0.29
Pipeline information fraction $I_{\Delta _t}$	0.3

For illustration purposes, we compare the designs using commonly used Pocock-like spending functions. In Table 4, we display the different boundary sets for the parameter configuration given in Table 3. Further, we present power, probability to stop for futility and expected sample size per arm. All of the designs are set up at a one-sided alpha level of $2.5\%$ and we use $\beta =0.2$ for the $\beta$-spending lower boundaries.

**Table 4:** Boundary sets $\{l_1, u_1, d_1, d_2\}$ and theoretical global performance characteristics for GSD, DR-GSD, and RR-GSD for the example in Mehta and Pocock [12], where $I_1 = 0.29$ and $I_{\Delta _t} = 0.3$

Method	$(l_1, u_1)$	$d_1$	$d_2$	*P*(*futility*)	$P(reject \, at \, interim)$	Power	$E_{\delta }[N]$
Fixed design	*NA*	*NA*	1.960	*NA*	*NA*	0.80	690
GSD	(0.259, 2.322)	*NA*	2.119	0.106	0.208	0.722	601.286
DR-GSD	(0.259, 2.322)	1.584	2.119	0.089	0.224	0.739	601.286
RR-GSD	$(-0.164, 1.815)$	1.960	2.043	0.098	0.329	0.737	569.222

Abbreviations: *GSD* Group-sequential design, *DR-GSD* Delayed response group-sequential design, *RR-GSD* Repeated rejection group-sequential design

The table indicates that the boundary sets of GSD and DR-GSD for the continuation region $(l_1, u_1)$ are equal, whereas those of RR-GSD differ. Specifically, the bounds are lower, making the recruitment more likely to stop for promising effects but less likely for non-promising effects upon observation of $n_1$ outcomes. Additionally, it is worth noting that the decision critical value of DR-GSD ($d_1=1.584$) is relatively small, attributed to the notably large amount of pipeline data. The power and expected sample size values of all designs are comparable and there is no method clearly superior to others. However, there is a slight power gain observed with the delayed response methods compared to the standard group-sequential method, without a noticeable increase in expected sample size. Note particularly that the probability to prove efficacy at interim is larger for the RR-GSD method due to the comparatively liberal upper recruitment stopping boundary, leading to a decrease in expected sample size.

## Discussion

Group-sequential designs are widely recognized for improving the flexibility and efficiency of clinical trial setups with respect to sample size, time and cost thereof. However, there has been limited attention given to handling patients recruited whose outcome measures are not immediately available during interim assessments. Our work aims at exploring to which degree group-sequential designs can enhance performance when accounting for data from delayed responses, rather than overlooking it as an inherent issue. Regarding performance evaluation, we argue that a meaningful performance comparison between group-sequential and delayed response group-sequential methods is best approached from a *global perspective*. This is because conditioning on interim results eliminates any differences, as long as the condition remains constant. Nevertheless, we introduce an adjusted version of the conditional performance score proposed by Herrmann et al. [24] in the appendix. While this score may not be an ideal tool for comparing standard group-sequential methods with delayed response group-sequential methods, it can still provide insights into overall performance by considering the location and variation of both conditional power and conditional expected sample size.

From a global performance evaluation perspective, the differences between the three candidate designs are negligible in most of the settings considered in this paper. While the global power has been found to differ at most by 2%, we note a considerably higher probability of rejecting the null hypothesis at interim with the RR-GSD. Therefore, if the choice of a trial design is made in realistic anticipation of early stopping, the RR-GSD may be deemed preferable. In terms of expected sample size, distinctions become apparent primarily with the RR-GSD as well. This observation can be attributed to its lower values for $u_1$ and $l_1$, resulting in a higher probability of halting recruitment when $Z_1 \ge u_1$, but a lower probability when $Z_1 \le l_1$. As a result, the expected sample size can exhibit lower or higher values compared to other methods, depending heavily on the true but in practice unknown effect size.

When drawing decisions about different clinical trial designs, it is crucial not only to assess their performance from a statistical point of view, but also to explore their practicality and logistical feasibility. To get a more holistic view on the applicability of the designs, operational aspects of those will be taken into account in the following.

Concerning software implementation, standard group-sequential designs constitute a well-established set of methods. Correspondingly, software packages such as rpact offer necessary tools for a hands-on implementation, planning and analysis of these designs. Additionally, the method proposed by Hampson and Jennison (2013) utilizing a binding error-spending recruitment stopping boundary, is mainly based on concepts from error-spending group-sequential designs. It deviates only through incorporation of the additional constraint needed to calculate the decision boundary. The DR-GSD has also been implemented for *K*-stage group-sequential designs in rpact, covering the implementation aspect but not extending to the analysis at this point. In contrast, for the delayed response group-sequential method with a nonbinding recruitment stopping boundary, the determination of the boundary set involves constraints slightly different from those used in standard group-sequential methods. Therefore, planning such a trial, especially with more than two stages, would require some implementation work. In summary, while tools for the execution and planning of standard group-sequential methods and the DR-GSD are readily available and diverse, the RR-GSD currently lacks a (commercial) software implementation. However, this design can easily be implemented using functionalities from rpact as well, as illustrated, for example, in the publicly available vignettes accessible through https://fpahlke.github.io/gsdwdr/ and https://www.rpact.org/vignettes/planning/rpact_delayed_response_designs/. When planning a delayed response group-sequential trial, several factors should be taken into consideration. At first, the efficacy of these designs depends on the amount of information available during interim analysis and the number of concurrent pipeline patients. This introduces a new planning parameter distinct from standard group-sequential methods. In practice, predicting the number of outstanding pipeline patients during interim analyses may be challenging. Therefore, trial planning should involve diverse scenarios, including various possible recruitment schemes (linear, constant, exponential) and different time behavior between recruitment and observations (constant, random). Simulations might provide a valuable tool to evaluate the robustness of delayed response designs in regards to a spectrum of possible sample sizes. The incorporation of a recruitment stopping decision leads to several challenges in the conduct. Communicating to halt a trial solely to draw a terminal decision on recruitment, not efficacy, might be challenging and there is a risk of misunderstanding the recruitment stopping as “premature efficacy or futility”. Consequently, care should be taken when interpreting interim results. Schüürhuis et al. [13] have addressed this concern by proposing a delayed response group-sequential method that allows for restarting the trial after having paused it, reducing the terminal nature of the recruitment stopping analysis. Secondly, the generally complicated study designs magnifies in large trials, particularly multicenter trials. For instance, managing parallel recruitment stopping poses a particular challenge, especially across multiple continents and time zones. Fortunately, error-spending methods offer flexibility by utilizing the actual trial information available, which may deviate from planning assumptions due to factors such as non-simultaneous stopping among participating centers, to establish decision boundaries.

Our paper started raising the question whether or not more complex designs accounting for delayed responses can be justified by a corresponding improvement in expected sample size and power. We found that regarding global performance, a notable gain in power is evident primarily in the probability of rejecting the null hypothesis at interim using the RR-GSD. Since this method also has a slightly lower expected sample size under the alternative and is designed to not allow for contradictory results - such as shifts from negative outcomes during recruitment stops to significant results at decision analysis - it may be considered the most recommendable method. Interestingly, however, the overall performance gain is limited, while no performance loss can be seen. This observation is valuable, allowing to shift the discussion of possible trial design choices from theoretical to practical properties as well. Finally, to address the question posed in the title, “What is the point of applying these methods?”, our response shall be as follows: While the performance gains from using delayed response models may be modest in the scenarios considered in here, the key advantages rather lie in regulatory and ethical considerations that support the use of delayed response group-sequential designs rather than simply discarding pipeline data. Additionally, in practice, delayed response designs can introduce a more structured decision-making process, reducing ambiguities during interim analyses. However, to fully realize these potential advantages, further research is required to effectively implement these methods, and practical experience is necessary to apply the designs effectively.

Our study also exhibits some limitations. At first, we restricted the designs under consideration to those with nonbinding lower boundaries to cover primarily designs of practical relevance. Moreover, we focused our analysis on continuous endpoints and designs featuring only one interim analysis. In the context of multistage designs, the influence of the number of pipeline patients on design performance and applicability may vary, especially with varying numbers of interim analyses. Furthermore, our focus was on designs based on standard error-spending group-sequential theory, given their widespread practical use. Other methodologies, including optimal designs, the incorporation of short-term endpoints for decision-making, and Bayesian approaches, represent potential directions for future research. Additionally, our comparison was centered on standard group-sequential methods ignoring pipeline patients, contrasted with methods that formally incorporate such data. This approach offers insights into the trade-off between neglecting and integrating pipeline data. Alternative perspectives, as demonstrated in Mukherjee et al. [15], could be evaluating the trade-off between disregarding the pipeline patients versus waiting for their data to become available for inclusion. This alternative approach naturally introduces an additional performance characteristic – the expected trial duration. Since the expected trial duration clearly depends on assumptions regarding recruitment pattern and delayed response behaviour, the inclusion of this parameter introduces a level of complexity that falls outside the scope of our current work.

## Supplementary Information

Supplementary Material 1.

## Data Availability

No datasets were generated or analysed during the current study. The software code to reproduce all results is available at https://fpahlke.github.io/gsdwdr/.
