# Supplementary material for: Two-stage group-sequential designs with delayed responses – what is the point of applying corresponding methods?
Source: BMC Med Res Methodol. 2024 Oct 17;24:242. doi: 10.1186/s12874-024-02363-7 (PMC11484224; doi:10.1186/s12874-024-02363-7)
Supplement: Supplementary file 1 — Supplementary Material 1. [file 12874_2024_2363_MOESM1_ESM.pdf]

Appendix for the manuscript "Two-stage group-sequential designs with delayed responses – what is the point of applying corresponding methods?"

Stephen Schüürhuis[1*], Gernot Wassmer[2], Meinhard Kieser[3], Friedrich Pahlke[2], Cornelia Ursula Kunz[4], Carolin Herrmann[1]

[1*]Institute of Biometry and Clinical Epidemiology, Charité - Universitätsmedizin Berlin, Corporate member of Freie Universität Berlin and Humboldt-Universität zu Berlin, Institute of Biometry and Clinical Epidemiology, Charitéplatz 1, Berlin, 10117, Germany.
[2]RPACT GbR, Am Rodenkathen 11, Sereetz, 23611, Germany.
[3]Institute of Medical Biometry, University Medical Center Ruprechts-Karls University Heidelberg, Im Neuenheimer Feld 130.3, Heidelberg, 69120, Germany.
[4]Biostatistics and Data Sciences, Boehringer Ingelheim GmbH & Co. KG, Birkendorfer Straße 65, Biberach an der Riß, 88400, Germany.

*Corresponding author(s). E-mail(s): stephen.schueuerhuis@charite.de;

Appendix A Considered spending functions for α and β

In this paper, we explore α- and β-spending functions, denoted as $a_{OF}(\cdot)$, $b_{OF}(\cdot)$, $a_P(\cdot)$, and $b_P(\cdot)$, to derive group-sequential boundary sets. The functions with the index OF represent O'Brien-Fleming-like spending functions, while those with the index P represent Pocock-like spending functions:

$$\begin{aligned}
a_{OF}(I) &= \min\left\{\mathbb{1}_{\{I>0\}} \cdot 2\left(1-\Phi\left(\frac{\Phi^{-1}(1-\frac{\alpha}{2})}{\sqrt{I}}\right)\right), \alpha\right\}, \\
b_{OF}(I) &= \min\left\{\mathbb{1}_{\{I>0\}} \cdot 2\left(1-\Phi\left(\frac{\Phi^{-1}(1-\frac{\beta}{2})}{\sqrt{I}}\right)\right), \beta\right\}, \\
a_P(I) &= \min\left\{\alpha(\ln(1+(e-1)I)), \alpha\right\}, \\
b_P(I) &= \min\left\{\beta(\ln(1+(e-1)I)), \beta\right\}.
\end{aligned}$$

Here, the function $\mathbb{1}_{\{I>0\}}$ is the indicator function is defined as

$$\mathbb{1}_{\{I>0\}} = \begin{cases} 1 & \text{if} \quad I > 0 \\ 0 & \text{otherwise.} \end{cases}$$

While the application of O'Brien-Fleming-like spending functions results in monotonically decreasing upper boundaries, the utilization of Pocock-like spending functions corresponds to approximately constant upper boundaries.

Appendix B Conditional performance measures

Conditional performance characteristics are based on conditioning on scenarios only where the interim test statistic suggest neither stopping early for efficacy nor for futility, but trial continuation. Hence, performance is conditioned on the interim results. A prominent example for a conditional performance criterion is the conditional power CP

$$CP_{\delta=\tilde{\delta}}(z_1) = 1 - \Phi\left(u_2 \cdot \frac{\sqrt{w_1^2 + w_2^2}}{w_2} - z_1 \cdot \frac{w_1}{w_2} - \tilde{\delta}\sqrt{\frac{n - n_1}{2}}\right),$$

where w_1 and w_2 are weights typically chosen proportional to the planned sample size, u_2 denotes the critical values, z_1 the observed test statistic at interim and $\tilde{\delta}$ some effect under which the conditional power should be evaluated. Moreover, $\Phi^{-1}(\,\cdot\,)$ denotes the cumulative distribution function of a standard normal distribution. Another example is the expected conditional sample size

$$\mathbb{E}[CN] = \mathbb{E}[N|Z_1 \in (l_1, u_1)],$$

where $N = n_1 + n_2(Z_1)$ denotes the random overall sample size. Note that the conditional sample size is a constant value in the case of classical group sequential trial designs since it is always the same number of patients that is recruited in the second stage if the trial continues. Especially for the conditional power it makes sense to also report a measure of variation next to describing its location since the conditional power value varies depending on the observed interim test statistic. This is also taken into account in the performance score by Herrmann et al. [24] The conditional performance score measures the performance using conditional power CP and conditional sample size CN both with respect to location e and variation v in dependence of an underlying effect size δ. The location components, denoted as $S_{e,CP}$ and $S_{e,CN}$ in the

following, describe the difference of the observed average conditional power or sample size compared to a target conditional power and sample size and set this in relation to the maximally possible deviation in conditional power or sample size:

$$S_{e,CP} = 1 - \frac{\mathbb{E}[CP(Z_1)] - CP_{target}}{CP_{max} - CP_{min}},$$
$$S_{e,CN} = 1 - \frac{\mathbb{E}[CN(Z_1)] - CN_{target}}{CN_{max} - CN_{min}}.$$

For the target values and maximally possible deviations $CP_{target}, CN_{target}, CP_{max}$ and CN_{max}, we refer to the table in the Appendix C. For the variation components $S_{v,CP}$ and $S_{v,CN}$, the observed variance of the conditional power or sample size is related to the maximally possible variance value (see Appendix C):

$$S_{v,CP} = 1 - \sqrt{\frac{Var(CP(Z_1))}{Var_{max}(CP(Z_1))}},$$
$$S_{v,CN} = 1 - \sqrt{\frac{Var(CN(Z_1))}{Var_{max}(CN(Z_1))}}.$$

Note that all score components are designed to range between $[0, 1]$, with higher values indicating performances that are closer to the optimal outcomes. Finally, the conditional performance score CS can be defined as a weighted sum of all components, that is

$$CS = w_{e,CN} \cdot S_{e,CN} + w_{v,CN} \cdot S_{v,CN} + w_{e,CP} \cdot S_{e,CP} + w_{v,CP} \cdot S_{v,CP},$$

where $w_{e,CN}, w_{v,CN}, w_{e,CP}, w_{v,CP}$ denote the weights for the different components. It is intuitive to use an equal weighting following, i.e., $w_{e,CN} = w_{v,CN} = w_{e,CP} = w_{v,CP} = 0.25$ but in principle a different weighting is also possible. Since all individual components are constructed such that a performance score value close to 0 refers

to a poor performance and a value close to 1 to an extraordinary performance, the same applies also to the overall performance score value. The conditional performance score is based on the expected values and variances of the random variables CP and CN, which inherit the randomness from Z_1. Given that no explicit closed-form expression for the distributions of CP and CN exists yet, the performance score must be calculated via simulation. An initial implementation of the conditional performance score is provided in the function `getPerformanceScore` within the `rpact` software package. This function takes a simulation result as input and outputs the components of the performance score.

Even though the conditional performance score was primarily defined for adaptive group sequential trial designs, it can also be applied to classic group sequential trial designs. As suggested in the manuscript, with some very minor modifications, the conditional performance score can also be applied to designs which account for delayed responses. We recommend only a comparison among binding futility stopping designs or among non-binding futility stopping designs since otherwise the condition underlying the conditional performance score is different. The minor modifications apply only to the reference values regarding the sample size where all n_1 values need to be replaced by $n_1 + n_{\Delta_t}$, i.e., the maximally possible difference in sample size becomes $n_{max} - (n_1 + n_{\Delta_t})$ instead of $n_{max} - n_1$ and the maximally possible variance as $((n_{max} - n_1 - n_{\Delta_t})/2)^2$ instead of $((n_{max} - n_1)/2)^2$. The power target values are exactly the same. A complete summary of the reference values for designs accounting for delayed responses can be found in the table in the Appendix C. Note that, as with the original performance score, the S_{CN} component is somewhat useless when only comparing classic group sequential trial designs among each other. On one hand, the variance component will always be a constant 1, as the second-stage sample size is fixed conditioned on proceeding to the second stage. Thus, it consistently achieves the optimal value of 1. For the location component, its value depends on the sample size

required for a fixed design, denoted as $n_{\text{fix},\delta}$ (see Appendix C). If $n_{\text{fix},\delta}$ exceeds $n_{\max}$, the location component will also be 1. However, if $n_{\text{fix},\delta} < n_{\max}$, the location component could become negative, particularly if $n_{\text{fix},\delta} < n_1 + n_{\Delta_t}$. To still leave room for a potential comparison with adaptive designs, however, we do not omit this component here. Further, note that the original definition of the conditional performance score was assuming immediate outcome measurements. However, when this is not the case, i.e. when pipeline data exist, the target value definition is naturally adapted. Hence, the only performance difference can be noted from the global perspective.

Appendix C Target values for the conditional performance score

Performance measure	$C{*}_{target}$ for $n_{fix,\delta} \leq n_{max}$ and $\delta \neq 0$	$C{*}_{target}$ for $n_{fix,\delta} > n_{max}$ or $\delta = 0$	$C{*}_{max} - C{*}_{min}$	Var_{max}
No pipeline data				
CN	$n_{fix,\delta}$	n_1	$n_{max} - n_1$	$((n_{max} - n_1)/2)^2$
CP	$1 - \beta$	α	$1 - \alpha$	$((1 - 0)/2)^2$
With pipeline data				
CN	$n_{fix,\delta}$	$n_1 + n_{\Delta_t}$	$n_{max} - (n_1 + n_{\Delta_t})$	$((n_{max} - n_1 - n_{\Delta_t})/2)^2$
CP	$1 - \beta$	α	$1 - \alpha$	$((1 - 0)/2)^2$

Appendix D Examples of boundaries for the four considered designs

Table D1: Boundary sets $\{l_1, u_1, d_1, d_2\}$ for DR-GSD, GSD, and RR-GSD design with $\alpha = 0.025$ and $\beta = 0.2$ for $\alpha-$ and $\beta-$spending functions a_{OF} and b_{OF} as well as a_P and b_P

Scenario	α-spending	β-spending	(I_1, I_2)	I_{Δ_t}	Method	(l_1, u_1)	d_1	d_2
1	a_{OF}	b_{OF}	$(0.3, 1)$	0.1	DR-GSD	$(-0.523, 3.929)$	1.940	1.960
					GSD	$(-0.523, 3.929)$	NA	1.960
					RR-GSD	$(-0.523, 3.928)$	1.960	1.960
2	a_{OF}	b_{OF}	$(0.4, 1)$	0.2	DR-GSD	$(0.0811, 3.357)$	2.025	1.962
					GSD	$(0.0811, 3.357)$	NA	1.962
					RR-GSD	$(0.080, 3.342)$	1.960	1.960
3	a_{OF}	b_{OF}	$(0.5, 1)$	0.3	DR-GSD	$(0.559, 2.963)$	2.074	1.969
					GSD	$(0.559, 2.963)$	NA	1.969
					RR-GSD	$(0.550, 2.895)$	1.960	1.965
4	a_P	b_P	$(0.3, 1)$	0.1	DR-GSD	$(0.305, 2.312)$	1.452	2.124
					GSD	$(0.305, 2.312)$	NA	2.124
					RR-GSD	$(0.137, 2.123)$	1.960	2.101
5	a_P	b_P	$(0.4, 1)$	0.2	DR-GSD	$(0.727, 2.224)$	1.656	2.165
					GSD	$(0.727, 2.224)$	NA	2.165
					RR-GSD	$(0.422, 1.859)$	1.960	2.090
6	a_P	b_P	$(0.5, 1)$	0.3	DR-GSD	$(1.083, 2.157)$	1.795	2.201
					GSD	$(1.083, 2.157)$	NA	2.201
					RR-GSD	$(0.680, 1.636)$	1.960	2.045

Abbreviations: GSD: Group-sequential design; DR-GSD: Delayed response group-sequential design; RR-GSD: Repeated rejection group-sequential design
